# Nano-enabled microbial bioinputs for sustainable agriculture: advances, mechanisms, applications, and future challenges

**DOI:** 10.3389/fpls.2026.1925672

**Published:** 2026-09-28

**Authors:** Isadora Camargo Pedrino, Paulo Teixeira Lacava

**Affiliations:** 1 Laboratory of Microbiology and Biomolecules, Department of Morphology and Pathology, Center for Biological and Health Sciences, Federal University of São Carlos (UFSCar), São Carlos, SP, Brazil; 2 Graduate Program in Biotechnology, Center for Exact Sciences and Technology, Federal University of São Carlos (UFSCar), São Carlos, SP, Brazil

**Keywords:** bio-based solutions, bionanofertilizers, bionanopesticides, microbial technologies, nano-enabled microbial bioinputs, sustainable agriculture

## Abstract

Microbial bioinputs have emerged as key components of sustainable agriculture owing to their ability to promote plant growth, improve nutrient acquisition, enhance tolerance to biotic and abiotic stresses, and suppress phytopathogens. Despite their considerable potential, the widespread adoption of these technologies remains constrained by limited environmental persistence, reduced formulation stability, and inconsistent performance under field conditions. In this context, nanotechnology has emerged as a promising approach to improve the stability, delivery efficiency, and agronomic reliability of microbial bioinputs. This review provides a critical and comprehensive assessment of recent advances in nano-enabled microbial bioinputs, discussing the mechanisms through which nanomaterials enhance microbial protection, stabilization, controlled release, delivery efficiency, and functional persistence. Particular emphasis is placed on bionanofertilizers and bionanopesticides as emerging technologies for improving nutrient use efficiency, biological crop protection, and soil–plant–microbiome interactions while reducing dependence on conventional agrochemicals. The review also critically examines current knowledge on biosafety, ecological impacts, regulatory frameworks, and the practical challenges associated with large-scale agricultural implementation. Current evidence indicates that nano-enabled formulations can address several limitations associated with conventional microbial bioinputs and contribute to more consistent agronomic performance under environmentally variable conditions. However, most available studies remain limited to laboratory and greenhouse experiments, while robust long-term validation under diverse field conditions is still lacking. In addition, uncertainties regarding environmental fate, nanoparticle–microbiome interactions, standardized biosafety assessment, scalable manufacturing, regulatory harmonization, and economic feasibility continue to constrain broader commercial adoption. Overall, nano-enabled microbial bioinputs represent a promising technological platform for bridging the gap between laboratory innovation and consistent field performance. Their successful implementation will depend on integrating advances in microbiology, nanotechnology, formulation engineering, and environmental risk assessment with rigorous field validation, scalable production strategies, and harmonized regulatory frameworks. Addressing these challenges will be essential to support the development of resilient, resource-efficient, and environmentally sustainable agricultural systems.

## 1 Introduction

Meeting the growing global demand for food while reducing the environmental footprint of agriculture has become one of the major challenges facing modern agricultural systems (Foyer et al., 2016). Although synthetic fertilizers and pesticides have contributed substantially to increasing agricultural productivity, their intensive use has also resulted in soil degradation, water contamination, biodiversity loss, and disruption of soil microbial communities (Vassilev et al., 2020; Gupta et al., 2022). At the same time, the increasing adoption of biological agricultural inputs and the establishment of regulatory frameworks supporting their development have reinforced the strategic importance of these technologies for the transition toward more sustainable agricultural systems (Backer et al., 2018; Government of Brazil, 2024).

Among these technologies, microbial bioinputs have emerged as key components of sustainable agricultural intensification strategies owing to their ability to promote plant growth, improve nutrient acquisition, enhance tolerance to biotic and abiotic stresses, and suppress plant pathogens. However, despite their demonstrated efficacy under controlled conditions, the inconsistent performance of microbial inoculants under field conditions remains one of the major barriers to their large-scale adoption. Limited formulation stability, low environmental persistence, sensitivity to abiotic fluctuations, and competition with native microbial communities frequently compromise microbial survival and functionality following application, resulting in variable agronomic performance (Backer et al., 2018; Kaminsky et al., 2019; Vassilev et al., 2020).

In this context, nanotechnology has attracted increasing attention as a promising strategy to overcome some of the major limitations affecting the field performance of microbial bioinputs. Owing to their unique physicochemical properties, nanostructured materials can enhance the protection, stability, establishment, and persistence of microbial components, thereby improving the potential effectiveness of these technologies in agricultural systems (Prasad et al., 2017; Dimkpa and Bindraban, 2018; Kah et al., 2018; de Moraes et al., 2021). The integration of microbial bioinputs with nanotechnology therefore represents a promising approach to improving the agronomic performance of biological solutions while preserving their environmental benefits.

This technological convergence has driven the development of distinct categories of nano-enabled microbial bioinputs, including nanoencapsulated microbial inoculants, microbial metabolites and microbial-derived products formulated within nanocarriers, systems based on the co-application of microorganisms and nanoparticles, and microbially synthesized nanoparticles (Mahanty et al., 2021; Kah et al., 2019). Although these approaches share the integration of nanotechnology with microbial components, they differ substantially in terms of composition, mechanisms of action, microbial viability requirements, biosafety profiles, regulatory considerations, and agricultural applications. Throughout this review, the term *nano-enabled microbial bioinputs* is used as an umbrella concept encompassing these distinct technological strategies, which are discussed as separate categories rather than equivalent formulations. Despite the remarkable advances achieved in recent years, challenges related to biosafety, the environmental fate of nanomaterials, formulation standardization, production scalability, regulatory approval, and agronomic validation under field conditions continue to limit their widespread implementation.

Against this background, this narrative review critically examines the current state of the art in nano-enabled microbial bioinputs by integrating the different technological strategies currently available and discussing their mechanisms of action, agricultural applications, advantages, limitations, biosafety considerations, regulatory challenges, and prospects for large-scale validation and implementation. By bringing these different dimensions together into a single critical analysis, this review aims to provide a comprehensive perspective on the current status of the field, identify the major scientific, technological, and regulatory challenges that continue to hinder the consolidation of these technologies, and highlight future research priorities required to bridge the gap between laboratory innovation and consistent agricultural application under real-world field conditions.

## 2 Literature search strategy

The literature considered in this structured narrative review was identified through searches of the Web of Science, Scopus, and Google Scholar databases, complemented by backward citation tracking of relevant publications. The literature search was last updated in July 2026 and employed combinations of the keywords *nano-enabled microbial bioinputs*, *microbial inoculants*, *biofertilizers*, *biopesticides*, *nanotechnology*, *nanoparticles*, *nanoformulation*, *encapsulation*, and *sustainable agriculture*, using the Boolean operators AND and OR.

Peer-reviewed articles published in English were prioritized, with emphasis placed on recent studies, although seminal publications were included when necessary to provide historical context and establish the conceptual framework of the review. Eligible studies included those describing the development, characterization, mechanisms of action, or agricultural applications of microbial bioinputs integrated with nanotechnology. Studies focusing exclusively on nanomaterials without microbial components or on non-agricultural applications were considered outside the scope of this review.

As this is a structured narrative review rather than a systematic review, the literature was selected to provide a comprehensive and critical overview of the field, and no formal systematic screening workflow or predefined number of retained studies was applied. The studies presented in **Tables 1**–**3** were selected based on their scientific relevance, representativeness of the different technological approaches encompassed by nano-enabled microbial bioinputs, originality, and contribution to illustrating the major advances, current limitations, and future perspectives discussed throughout this review. Accordingly, these tables were designed to provide representative examples of the current state of the field rather than a systematic or exhaustive compilation of all available studies.

**Table 1:** Comparison of the main nanomaterials employed in bio-nanofertilizer formulations.

Nanomaterial	Main properties	Advantages	Limitations	Representative applications	References
Silica nanoparticles	High surface area, tunable porosity, mesoporous structure, high physicochemical stability, and versatile surface functionalization.	Efficient encapsulation of microorganisms and bioactive compounds; controlled release; protection against environmental stresses; improved formulation stability and nutrient-use efficiency.	Performance depends on particle size, morphology, surface chemistry, and aggregation state. Long-term persistence, soil transformation, and interactions with non-target microorganisms require further investigation.	Microbial encapsulation, controlled nutrient delivery, seed coating, and bio-nanofertilizers.	Jeelani et al. (2020); Janjua et al. (2023); Naaz et al. (2023).
Nanoclays	Layered phyllosilicates with high surface area, high cation exchange capacity, natural abundance, and versatile surface modification.	Efficient encapsulation and sustained release of microorganisms and bioactive compounds; improved nutrient- and water-use efficiency; environmentally friendly carrier systems.	Environmental behavior depends on clay composition, particle size, surface modification, and interactions with soil components. Long-term environmental fate and ecotoxicological effects remain insufficiently understood.	Microbial inoculants, controlled nutrient delivery, seed coating, and bio-nanofertilizers.	Merino et al. (2020); Rao et al. (2024); Amin et al. (2025).
Hydrogels	Three-dimensional crosslinked polymeric networks with high water retention, tunable porosity, biodegradability, biocompatibility, and controlled-release capability.	Improved moisture retention; controlled release of microorganisms, nutrients, and bioactive compounds; enhanced formulation stability and drought tolerance.	Performance depends on polymer composition, degradation behavior, and environmental conditions. Long-term stability and degradation under field conditions require further investigation.	Microbial carriers, controlled-release biofertilizers, seed coating, soil conditioners, and drought mitigation.	Kaur et al. (2023); Vedovello et al. (2024); Qin et al. (2024).
Liposomal systems	Phospholipid vesicles capable of encapsulating hydrophilic and hydrophobic compounds with tunable composition and surface properties.	Efficient encapsulation and protection of microorganisms and bioactive compounds; controlled release; improved stability, bioavailability, and biocompatibility.	Performance depends on lipid composition, formulation stability, and environmental conditions. Stability and degradation under agricultural conditions remain insufficiently characterized.	Delivery of beneficial microorganisms and bioactive compounds, seed coating, and controlled-release biofertilizers.	Kyrychenko and Kovalenko (2025); Sharmila et al. (2025); Kutasy-Takács et al. (2026).
Carbon-based nanomaterials	Carbon nanostructures with high surface area, mechanical strength, tunable surface chemistry, and unique physicochemical properties.	Efficient delivery of nutrients and bioactive compounds; controlled release; enhanced plant growth and stress tolerance.	Performance depends on nanomaterial type, functionalization, size, dosage, and exposure conditions. Potential phytotoxicity and long-term impacts on soil microbial communities require further investigation.	Delivery of nutrients and bioactive compounds, microbial formulations, and bio-nanofertilizers.	Husen (2020); Zhu et al. (2022); Aacharya and Chhipa (2020); Karuvelan et al. (2025).
Functionalized biopolymers	Renewable and biodegradable polymeric matrices (e.g., chitosan, alginate, cellulose, starch, pectin, gelatin, and cyclodextrins) with tunable physicochemical properties and controlled-release capability.	Efficient encapsulation of microorganisms and bioactive compounds; controlled release; high biocompatibility and biodegradability; improved formulation stability and environmental sustainability.	Performance depends on polymer composition, formulation design, degradation behavior, and environmental conditions. Long-term stability and degradation under field conditions require further investigation.	Microbial encapsulation, controlled-release biofertilizers, seed coating, hydrogels, and soil conditioners.	Gamage et al. (2022); Saberi Riseh et al. (2024); Liyakat (2024).

**Table 2:** Representative studies on nano-enabled microbial bioinputs for biological control and plant growth promotion.

Biological component	Bioinput category	Nano-enabled strategy	Nanotechnology platform	Experimental setting	Plant system	Main outcome	Reference
*Rhizobium tropici*	Living microbial inoculant	Co-application with nanoparticles	Fe3O4 nanoparticles	Growth chamber	Common bean (*Phaseolus vulgaris*)	Enhanced nodulation, biological nitrogen fixation and iron uptake.	De Souza-Torres et al., 2021
PGPR consortium	Living microbial inoculant	Co-application with nanofertilizer	ZnO nanoparticles	Greenhouse (pot)	Wheat (*Triticum aestivum*)	Improved plant growth, nutrient uptake and tolerance to salinity stress.	Jalal et al., 2023
*Acinetobacter pittii* + *Bacillus velezensis*	Living microbial inoculant	Co-application with nanoparticles	Green-synthesized ZnS nanoparticles	Greenhouse (pot)	Tomato (*Solanum lycopersicum*)	Reduced root rot severity and enhanced antioxidant defense, nutrient uptake, growth and yield.	Shah et al., 2023
*Bacillus* culture filtrate	Microbial-derived bioactive product	Co-application with nanoparticles	ZnO nanoparticles	Laboratory and greenhouse	Cucumber (*Cucumis sativus*)	Reduced bacterial leaf spot severity and improved plant physiology and fruit yield.	Helaly et al., 2026
*Bacillus subtilis* Vru1	Living microbial inoculant	Polymeric nanoencapsulation	Alginate–bentonite–starch matrix enriched with TiO2 nanoparticles	Laboratory and greenhouse	Common bean (*Phaseolus vulgaris*)	Improved bacterial survival and root colonization, reduced *Rhizoctonia* root rot and promoted plant growth.	Saberi Riseh & Moradi Pour, 2020
*Azospirillum brasilense* Ab-V5	Living microbial inoculant	Polymeric nanoencapsulation	Polymeric matrix (PGCit) with trehalose	Laboratory	Maize (*Zea mays*)	Improved storage stability and recovery of viable cells after seed coating.	Alves et al., 2026
*Pseudomonas fluorescens*	Living microbial inoculant	Polymeric nanoencapsulation	Chitosan-based nanocapsules	Pot and field	Tomato (*Solanum lycopersicum*)	Increased shelf life, improved soil fertility and enhanced tomato growth and yield under pot and field conditions.	Beula Isabel et al., 2024

AgNPs, silver nanoparticles; Fe₃O₄, magnetite nanoparticles; PGCit, poly(glycerol citrate); PGPR, plant growth-promoting rhizobacteria; TiO_2_, titanium dioxide; ZnO, zinc oxide; ZnS, zinc sulfide. Experimental setting refers to the highest level of validation reported in each study (laboratory, greenhouse/pot, growth chamber, or field).

**Table 3:** Representative experimental evidence illustrating the context-dependent ecological effects of engineered nanoparticles on beneficial soil microorganisms and non-target organisms and their implications for the development of nano-enabled microbial bioinputs.

Nanomaterial	Biological target	Main findings	Key implication for nano-enabled microbial bioinputs	Reference
ZnO nanoparticles	*Rhizobium leguminosarum*–legume symbiosis	Delayed nodulation, reduced establishment of biological nitrogen fixation, premature nodule senescence, and morphological alterations in rhizobial cells.	Nanoparticles may interfere with beneficial plant–microbe interactions, highlighting the importance of assessing the compatibility between nanomaterials and microbial inoculants before field application.	Huang et al. (2014)
ZnO and CeO_2_ nanoparticles	Soybean–rhizobia symbiosis	ZnO nanoparticles primarily promoted Zn accumulation in plant tissues, whereas CeO_2_ nanoparticles impaired biological nitrogen fixation and nodule functionality.	Ecological responses are nanoparticle-specific and cannot be generalized across different nanomaterials, even under similar experimental conditions.	Priester et al. (2012)
Ag nanoparticles	Arbuscular mycorrhizal fungi (AMF)	Reduced AMF diversity, root colonization, alkaline phosphatase activity, and phosphorus acquisition.	Nanoparticles may impair soil ecosystem functions mediated by beneficial microorganisms, emphasizing the need to evaluate functional endpoints in addition to microbial abundance.	Cao et al. (2017)
TiO_2_ nanoparticles	Soil bacterial communities	Bacterial community composition changed according to TiO_2_ nanoparticle concentration and exposure time, whereas community functional profiles remained largely unaffected, suggesting functional redundancy.	Microbial responses to nanoparticles are strongly influenced by exposure conditions and should be evaluated using both taxonomic and functional endpoints.	Zhai et al. (2019)
Ag nanoparticles	*Eisenia fetida* (earthworm)	Altered gut microbial community composition and trace-element accumulation; adverse effects were mitigated in substrates with higher organic matter content.	Environmental conditions, particularly soil organic matter, can substantially reduce nanoparticle bioavailability and toxicity toward non-target organisms, highlighting the importance of realistic exposure scenarios.	Zapałowska et al. (2026)

AMF, arbuscular mycorrhizal fungi. Representative experimental studies demonstrating that the ecological effects of engineered nanoparticles on beneficial microorganisms and non-target soil organisms depend on nanoparticle composition, biological target, exposure conditions, and environmental characteristics. Collectively, these findings highlight the need for context-specific ecotoxicological assessments before the large-scale agricultural application of nano-enabled microbial bioinputs.

## 3 Microbial bioinputs: opportunities and limitations under field conditions

Microbial bioinputs have emerged as key components of sustainable agricultural systems due to their ability to promote plant growth, improve nutrient acquisition, enhance tolerance to biotic and abiotic stresses, and suppress plant pathogens. Endophytic and rhizospheric microorganisms contribute to plant development through a wide range of mechanisms, including biological nitrogen fixation, nutrient mobilization, phytohormone production, siderophore synthesis, and activation of plant defense responses, resulting in increased crop productivity, improved nutrient use efficiency, enhanced soil health, and greater agroecosystem resilience (Adeleke et al., 2022; Backer et al., 2018; Hardoim et al., 2015; Olanrewaju et al., 2017). Among the most extensively studied microbial genera are *Azospirillum*, *Bacillus*, *Pseudomonas*, *Rhizobium*, *Serendipita*, and *Trichoderma*, which have been widely investigated for their roles in plant growth promotion, nutrient mobilization, stress mitigation, and pathogen suppression (Orozco-Mosqueda et al., 2022).

Microbial bioinputs used in agriculture are traditionally classified into two major categories: biofertilizers and microbial biocontrol agents. Biofertilizers promote plant nutrition and growth through biological nutrient acquisition processes, whereas microbial biocontrol agents reduce disease incidence through mechanisms such as direct antagonism, competition for ecological niches, production of antimicrobial metabolites, and induction of plant defense responses (Vassilev et al., 2020; El-Saadony et al., 2022; Bonaterra et al., 2022). Collectively, these functions contribute to reducing the dependence on synthetic fertilizers and pesticides, thereby supporting more sustainable, resilient, and environmentally responsible agricultural systems.

Despite the considerable progress achieved over the past decades, the large-scale adoption of microbial bioinputs remains constrained by the variability of their performance under field conditions. Although numerous studies have demonstrated high efficacy under laboratory and greenhouse conditions, the survival, establishment, and functionality of inoculated microorganisms following application are strongly influenced by factors such as formulation stability, environmental conditions, microbial persistence, colonization efficiency, and interactions with native soil microbial communities. Consequently, the benefits observed under controlled conditions are not always reproduced in agricultural environments, compromising the consistency of agronomic responses, the reliability of these technologies, and their widespread adoption (Backer et al., 2018; Kaminsky et al., 2019; Vassilev et al., 2020).

Overcoming these limitations has become one of the primary goals of research on microbial bioinput development. In this context, a variety of advanced formulation strategies have been investigated to improve microbial protection, enhance storage stability, facilitate establishment following inoculation, and prolong microbial persistence and functionality under field conditions. Among these strategies, the integration of nanotechnology with microbial bioinputs has attracted increasing attention by offering new opportunities to improve the protection of biological agents, modulate their release, enhance their stability, and increase the consistency of agronomic performance across diverse cropping systems (Vassilev et al., 2020; Kah et al., 2019; de Moraes et al., 2021). The following sections examine the principal nanotechnology-based approaches currently employed in the development of nano-enabled microbial bioinputs, highlighting their formulation strategies, mechanisms of action, agricultural applications, advantages, limitations, and the remaining challenges for their large-scale implementation.

## 4 From agricultural nanotechnology to nano-enabled microbial bioinputs

Nanotechnology has significantly expanded the possibilities for developing more efficient agricultural inputs, driving advances in plant nutrition, crop protection, and, more recently, the formulation of microbial bioinputs. Properties such as high surface area, tunable surface reactivity, and controlled-release capabilities provide nanomaterials with remarkable versatility for optimizing the delivery of nutrients and bioactive compounds, reducing environmental losses, and improving the performance of agricultural inputs compared with conventional formulations (Kah et al., 2018; Zulfiqar et al., 2019). As a result, nanotechnology has become a strategic platform for simultaneously enhancing agronomic efficiency and the sustainability of agricultural production systems.

Among the most established applications of agricultural nanotechnology are nanofertilizers and nanopesticides. Nanofertilizers have been developed to improve nutrient availability, retention, and uptake efficiency, thereby supporting more sustainable fertilization strategies. Nanopesticides, in turn, are designed to enhance the stability of active compounds, optimize their delivery, and reduce the environmental impacts associated with conventional pesticide formulations. In both cases, technologies such as nanoencapsulation and controlled- or stimuli-responsive delivery systems protect active compounds from premature degradation, regulate their availability, and enable their release in response to specific environmental or biological cues (Arora, 2024; Behl et al., 2024).

The advances achieved with these platforms have established the scientific and technological foundation for extending nanotechnology to other categories of agricultural inputs. Within this context, the development of nano-enabled microbial bioinputs represents a natural progression in the application of nanotechnology to agriculture, incorporating well-established concepts such as nanoencapsulation, carrier systems, and controlled-release technologies into formulations containing microorganisms or microbial-derived products. In these systems, nanomaterials perform functions that extend beyond the simple transport of active ingredients, serving as multifunctional platforms capable of protecting biological agents, modulating their release, promoting their establishment at the soil–plant interface, and enhancing formulation stability under agricultural conditions (Kah et al., 2018; Dimkpa and Bindraban, 2018; de Moraes et al., 2021; Mahanty et al., 2021; Mgadi et al., 2024; Díaz-Rodríguez et al., 2025).

As a consequence of this technological evolution, bionanofertilizers and bionanopesticides have emerged as two of the most important applications of nano-enabled microbial bioinputs. These platforms integrate living microorganisms or microbial-derived products with nanostructured materials through diverse formulation strategies to improve nutrient delivery, crop protection, and the persistence of biological agents, representing one of the most promising frontiers of agricultural nanotechnology (de Moraes et al., 2021; Mahanty et al., 2021; Behl et al., 2024).

Despite the growing body of evidence supporting the potential of these technologies, their agronomic performance remains highly dependent on multiple factors, including the composition, size, and surface functionalization of nanomaterials, their compatibility with microbial strains, formulation design, environmental conditions, and application strategy. Furthermore, most available evidence still derives from laboratory, greenhouse, or short-term experiments, whereas robust long-term field validation and commercial-scale assessments remain limited. In addition, biosafety concerns, the environmental fate of nanomaterials, interactions with native microbiomes, regulatory standardization, production scalability, and formulation reproducibility continue to represent major challenges to the widespread adoption of these technologies (Kah et al., 2019; Mgadi et al., 2024; El-Saadony et al., 2022).

Overall, the evolution of agricultural nanotechnology demonstrates how strategies originally developed for fertilizers and pesticides are now being adapted to improve microbial bioinputs. This convergence of nanotechnology and agricultural microbiology expands the opportunities for developing more efficient and robust biological formulations while highlighting the need for integrated advances in formulation engineering, microbiology, biosafety, environmental risk assessment, large-scale manufacturing, and field validation to support the safe and effective implementation of these technologies in sustainable agricultural systems.

## 5 Bionanofertilizers: integrating microbial and nanotechnological approaches for plant growth promotion

Bionanofertilizers represent one of the major applications of nano-enabled microbial bioinputs discussed in this review. In this context, diverse nanoformulation strategies, including nanoencapsulation, controlled-release systems, nanostructured carriers, and hybrid formulations, have considerably expanded the possibilities for developing more efficient biological fertilization systems by improving the performance of plant growth-promoting microorganisms (PGPMs) and microbial-derived bioactive products under agricultural conditions (Mahanty et al., 2021; Arora, 2024). Beyond protecting biological components from environmental factors that compromise their viability and functionality, these technologies enable the controlled release of nutrients and bioactive compounds, prolong their persistence following application, and promote more efficient interactions among microbial inoculants, plant root systems, and the soil microbiome (Behl et al., 2024; de Moraes et al., 2021).

The effectiveness of these formulation strategies is closely associated with the physicochemical properties of the nanomaterials employed. The growing diversity of available nanomaterials has substantially expanded the range of delivery platforms that can be tailored to the characteristics of biological components and the specific objectives of each formulation (Mahanty et al., 2021; Arora, 2024; Behl et al., 2024). Among the most extensively investigated materials are silica nanoparticles, nanoclays, hydrogels, liposomal systems, carbon-based nanomaterials, and functionalized biopolymers, whose properties directly influence encapsulation efficiency, formulation stability, release kinetics, and the agronomic performance of microbial bioinputs (Jeelani et al., 2020; Husen, 2020; Gamage et al., 2022; Kaur et al., 2023; Janjua et al., 2023; Naaz et al., 2023; Saberi Riseh et al., 2024; Qin et al., 2024; Vedovello et al., 2024; Amin et al., 2025; Kyrychenko and Kovalenko, 2025; Kutasy-Takács et al., 2026).

However, nanomaterial selection determines not only formulation performance but also biosafety profiles and environmental behavior. Biopolymer-based materials, hydrogels, and lipid-based carriers have attracted particular attention because of their high biocompatibility and capacity to provide controlled release, although further studies are needed to evaluate their stability and degradation under field conditions. In contrast, metallic and metal oxide nanoparticles require more careful assessment because of their potential to generate reactive oxygen species, release metal ions, and exhibit antimicrobial activity, all of which may affect non-target organisms depending on exposure conditions (Ge et al., 2011, 2012; Yamini et al., 2023). Likewise, silica-based materials, nanoclays, and carbon-based nanomaterials differ in their stability, persistence, and interactions with soil environments, resulting in distinct environmental behaviors among material classes (Jeelani et al., 2020; Husen, 2020; Zhu et al., 2022; Merino et al., 2020; Janjua et al., 2023; Naaz et al., 2023; Amin et al., 2025). Consequently, nanomaterial selection should simultaneously consider delivery efficiency, compatibility with biological components, and potential environmental impacts to support the development of both effective and safe formulations (Kah et al., 2019; Mgadi et al., 2024). These differences are summarized in **Table 1**, which compares the principal classes of nanomaterials used in bionanofertilizer formulations, highlighting their properties, advantages, limitations, representative applications, and biosafety considerations.

Current evidence demonstrates that different nanoformulation strategies consistently enhance plant growth promotion and, in many cases, also improve biological disease control. These findings, summarized in **Table 2**, encompass both living microbial inoculants and microbial-derived bioactive products and primarily involve two nanotechnological approaches: co-application with nanoparticles or nanofertilizers and polymeric nanoencapsulation (Saberi Riseh & Moradi Pour, 2020; De Souza-Torres et al., 2021; Jalal et al., 2023; Shah et al., 2023; Beula Isabel et al., 2024; Helaly et al., 2026; Alves et al., 2026). Despite the diversity of biological components, nanomaterials, and plant systems investigated, the available studies consistently report common benefits, including improved microbial survival and viability (Saberi Riseh & Moradi Pour, 2020; Alves et al., 2026), enhanced root colonization (Saberi Riseh & Moradi Pour, 2020), increased nutrient uptake (De Souza-Torres et al., 2021; Jalal et al., 2023; Shah et al., 2023), greater tolerance to biotic and abiotic stresses (Jalal et al., 2023; Shah et al., 2023), reduced disease severity (Saberi Riseh & Moradi Pour, 2020; Shah et al., 2023; Helaly et al., 2026), and improved plant growth, development, and productivity (De Souza-Torres et al., 2021; Jalal et al., 2023; Shah et al., 2023; Beula Isabel et al., 2024; Helaly et al., 2026). Although most of the available evidence still derives from laboratory, growth chamber, and greenhouse experiments, recent field studies reinforce the potential of these technologies for commercial agricultural applications (Beula Isabel et al., 2024). At the same time, the considerable diversity of biological components, nanomaterials, formulation strategies, and experimental conditions limits direct comparisons among studies and highlights that optimization must be tailored to each specific agricultural system (Mishra et al., 2021; Behl et al., 2024).

Overall, the available evidence indicates that bionanofertilizers represent a promising strategy for improving the efficiency and reliability of microbial bioinputs for plant growth promotion. Nevertheless, further advances in formulation engineering, methodological standardization, biosafety assessment, field validation, and large-scale production will be essential to establish these technologies as viable and sustainable solutions for modern agriculture.

## 6 Bionanopesticides: integrating microbial and nanotechnological approaches for sustainable crop protection

Bionanopesticides represent another major application of nano-enabled microbial bioinputs discussed in this review, integrating beneficial microorganisms or microbial-derived products with nanostructured materials to improve the stability, efficacy, and reliability of biological plant disease control. By combining the antagonistic activity of microbial biocontrol agents with the protective and controlled-release properties provided by nanomaterials, these formulations seek to overcome important limitations associated with conventional microbial products, including reduced persistence under adverse environmental conditions, sensitivity to ultraviolet radiation and desiccation, and inconsistent performance under field conditions (Kah et al., 2018; Mishra et al., 2021; Anand et al., 2023).

Nanoencapsulation and nanomaterial-assisted delivery systems constitute the core of these formulations. Different nanostructured platforms can protect microbial cells and bioactive metabolites from environmental degradation, enhance their adhesion to plant surfaces, facilitate microbial establishment following application, and regulate the controlled release of active compounds, thereby prolonging their biological activity under agricultural conditions (Pinto et al., 2022; Mishra et al., 2021; Behl et al., 2024). Beyond delivering viable microorganisms, nanoformulation strategies have also been widely explored for transporting microbial metabolites and other bioactive compounds with antimicrobial activity or plant defense-inducing properties. By increasing the stability of these compounds, protecting them from environmental degradation, and regulating their release over time, nanoformulations can improve the availability of active agents at the appropriate time and site of action, contributing to the development of more efficient and sustainable biological control systems (Mishra et al., 2021; Behl et al., 2024).

Current evidence indicates that different nanoformulation strategies can enhance the performance of microbial biocontrol agents through complementary mechanisms, including improved protection of biological components, prolonged persistence after application, and stronger interactions with the host plant. As summarized in **Table 2**, studies involving both living microbial inoculants and microbial-derived products have consistently reported promising results, including reduced disease severity, enhanced stability of biological agents, and improved agronomic performance (Saberi Riseh & Moradi Pour, 2020; Shah et al., 2023; Beula Isabel et al., 2024; Helaly et al., 2026; Alves et al., 2026). However, the considerable diversity of nanomaterials, biological components, target pathogens, and experimental conditions limits direct comparisons among studies and highlights that formulation optimization must be tailored to specific agricultural systems (Mishra et al., 2021; Behl et al., 2024).

Beyond directly improving formulation stability, biological mechanisms associated with microbial establishment may also contribute to the effectiveness of bionanopesticides. Enhanced survival of microbial agents following application can promote more successful rhizosphere colonization, facilitating niche occupation and stronger interactions with the host plant. In this context, biofilm formation plays a central role in stable root colonization. Biofilms consist of microbial communities embedded within an extracellular polymeric substances (EPS) matrix that promotes adhesion to root surfaces, protects microbial cells against environmental stresses, and enhances their persistence in the rhizosphere, thereby sustaining antagonistic activity throughout the crop cycle (Bais et al., 2004; Flemming et al., 2016; Flemming and Wuertz, 2019; Brokate et al., 2024).

The formation and maintenance of biofilms depend on complex cell-to-cell communication mechanisms, among which quorum sensing plays a key role in coordinating gene expression related to cell adhesion, extracellular matrix production, and collective microbial behavior (Mukherjee and Bassler, 2019; Flemming and Wuertz, 2019). Because nanoformulations enhance microbial survival and establishment following application, it has been proposed that these technologies may also indirectly influence quorum sensing-regulated processes. Some studies suggest that specific nanomaterials can modify the microbial microenvironment or alter the local availability of signaling molecules, potentially affecting biofilm formation and antagonistic activity (Behl et al., 2024). Nevertheless, the available evidence remains limited and is derived predominantly from *in vitro* studies or experimental systems distinct from plant–microbe interactions. Therefore, although these interactions represent a promising mechanistic perspective for understanding bionanopesticide performance, their relevance under agricultural conditions still requires experimental validation.

Despite the significant progress achieved in recent years, the widespread implementation of bionanopesticides remains constrained by important scientific, technological, and regulatory challenges. Considerable heterogeneity in nanomaterials, biological agents, formulation methods, application protocols, and efficacy assessment criteria complicates comparisons among studies and limits the identification of the factors most strongly associated with formulation performance (Kah et al., 2018; Mishra et al., 2021; Behl et al., 2024). Furthermore, most of the available evidence still derives from laboratory and controlled-environment experiments, whereas field validation and large-scale agricultural evaluations remain scarce (Anand et al., 2023; Behl et al., 2024). As highlighted by Kah et al. (2018), studies directly comparing nanoformulations with their conventional counterparts are still limited, making it difficult to establish the actual benefits provided by nano-enabled delivery systems. Considering the complexity of interactions among plants, microorganisms, nanomaterials, and environmental factors, long-term field studies are essential to confirm the stability, efficacy, and reproducibility of these technologies under diverse agricultural conditions.

At the same time, emerging nanostructured platforms continue to expand the possibilities for bionanopesticide development. Among the most promising approaches are stimuli-responsive nanoformulations, which are capable of modulating the release of microorganisms or bioactive compounds in response to environmental cues such as changes in pH, temperature, or other stimuli associated with the plant microenvironment (Behl et al., 2024; Kyrychenko and Kovalenko, 2025). Although these systems represent an important technological advance, their practical implementation still faces challenges related to large-scale production, preservation of biological component stability during processing and storage, and the development of economically viable manufacturing processes.

In addition to technological challenges, biosafety and regulatory issues remain fundamental to the future adoption of bionanopesticides. Although nanoformulations may alter the environmental behavior of active ingredients, comprehensive studies integrating agronomic performance, environmental exposure, and ecological impacts under realistic agricultural conditions remain scarce. Lead et al. (2018) emphasized that, despite significant advances in understanding the environmental fate, transformation, bioavailability, and toxicity mechanisms of nanomaterials, substantial knowledge gaps persist regarding environmental exposure assessment and the behavior of different nanomaterials in terrestrial and aquatic ecosystems. From a sustainable development perspective, Yin et al. (2018) argued that nanotechnology-based agricultural products should incorporate integrated assessments of life cycle, environmental exposure, ecological impacts, and agronomic benefits from the earliest stages of development. Accordingly, the establishment of standardized risk assessment methodologies and the development of dedicated regulatory frameworks will be essential to ensure the safe, effective, and sustainable implementation of bionanopesticides.

## 7 Comparative advantages, current limitations, and field implementation

Although nano-enabled microbial bioinputs have demonstrated significant potential to improve the stability and performance of microbial formulations, their ultimate value for agriculture will depend on whether these technological advances can be translated into consistent agronomic benefits under real field conditions. In this context, a critical assessment of their comparative advantages, current limitations, biosafety implications, and implementation prospects is essential to determine their potential as a next-generation technological platform for sustainable agriculture.

Compared with both conventional microbial bioinputs and synthetic nanofertilizers and nanopesticides, nano-enabled microbial bioinputs uniquely combine the functional properties of nanomaterials with the biological mechanisms associated with beneficial microorganisms. This integration can enhance formulation stability, protect microorganisms and microbial-derived bioactive compounds from environmental stress, modulate their release, and promote more effective interactions with the host plant. Nevertheless, the performance of these formulations is influenced by multiple factors, including nanomaterial composition and physicochemical properties, compatibility with the biological component, soil characteristics, environmental conditions, and application strategy, highlighting the highly context-dependent nature of these systems (Zuverza-Mena et al., 2017; Mgadi et al., 2024). This complexity limits the identification of universally applicable strategies and emphasizes the need to develop formulations tailored to specific edaphoclimatic conditions and agricultural production systems rather than assuming that a single technology will perform consistently across diverse environments (Vaidya et al., 2024, 2016; Mgadi et al., 2024).

Despite these advantages, several limitations continue to restrict large-scale implementation. Although an increasing number of studies have reported promising results, field validation remains relatively limited, and agronomic gains are not always proportional to the added complexity or production costs of nanoformulations. Furthermore, incorporating living microorganisms introduces additional challenges related to microbial viability, formulation shelf life, storage stability, and interactions with native soil microbial communities, all of which require further optimization before widespread commercial adoption (Singh and Kumar, 2024; Samantaray et al., 2024; Monica et al., 2025).

Another important consideration concerns the potential ecotoxicological effects of nanomaterials on non-target organisms. Although inorganic nanoparticles with antimicrobial properties, such as silver (Ag), zinc oxide (ZnO), and titanium dioxide (TiO_2_), may contribute to pathogen suppression, they may also affect beneficial soil microorganisms, including plant growth-promoting bacteria, arbuscular mycorrhizal fungi, and other members of the soil microbiome involved in essential processes such as biological nitrogen fixation, mycorrhizal colonization, nutrient cycling, and maintenance of microbial community structure. However, current evidence indicates that these effects are not an intrinsic characteristic shared by all nanomaterials but instead depend on the interaction between nanomaterial physicochemical properties, the exposed organisms, and environmental conditions (Tian et al., 2019; Chen et al., 2024). These representative experimental findings are summarized in **Table 3**, illustrating how different nanomaterials produce distinct responses depending on the biological system and exposure conditions.

This context dependency is further reinforced by the fact that the environmental fate and bioavailability of nanoparticles are strongly influenced by soil physicochemical properties, including pH, organic matter content, mineral composition, moisture, and interactions with native microbial communities. These factors can substantially modify nanoparticle mobility, persistence, bioavailability, and biological activity compared with controlled laboratory conditions (Cornelis et al., 2014; de Moraes et al., 2021; Yamini et al., 2023; Chen et al., 2024). Likewise, non-target organisms such as earthworms, insects, and aquatic organisms exposed through runoff may respond differently depending on nanomaterial composition, concentration, persistence, and environmental fate. Consequently, results obtained under laboratory or greenhouse conditions cannot always be directly extrapolated to agricultural systems under field conditions, highlighting the need for standardized ecotoxicological assessments and long-term field studies to support the environmentally responsible implementation of nano-enabled microbial bioinputs (Jeelani et al., 2020; Janjua et al., 2023; Saberi Riseh et al., 2024).

From a practical perspective, nano-enabled microbial bioinputs should not be regarded as a universal solution for all agricultural systems. Their greater technological complexity and development costs require clear agronomic advantages, economic feasibility, scalability, and consistent performance under realistic production conditions to justify their adoption (Mahanty et al., 2021; Behl et al., 2024; Mgadi et al., 2024). Accordingly, these technologies are expected to be particularly advantageous in environments subjected to biotic or abiotic stresses, nutrient-deficient soils, degraded agricultural lands, and low-input production systems, where conventional microbial inoculants often exhibit limited persistence or inconsistent performance (Mahanty et al., 2021; Behl et al., 2024). Conversely, their adoption in highly intensive agricultural systems will depend on demonstrating clear improvements in productivity, cost-effectiveness, and operational reliability under commercial production conditions (Mgadi et al., 2024).

In addition to scientific, technological, and economic challenges, differences in regulatory frameworks among the world’s major agricultural regions represent important barriers to the international commercialization of nano-enabled microbial bioinputs. The European Union has established harmonized regulations with stringent requirements for biostimulants and biopesticides, whereas regulatory oversight in the United States is distributed among different federal agencies according to product classification. In contrast, regulatory systems in several Asian countries are still evolving to keep pace with advances in emerging biological technologies. These differences influence product registration requirements, approval timelines, market access, and technology transfer, frequently delaying the international commercialization of innovative microbial formulations (Vermelho et al., 2026).

Consequently, future progress will require coordinated advances in formulation engineering, biosafety assessment, international regulatory harmonization, manufacturing standardization, and long-term field validation to ensure both agronomic effectiveness and environmental sustainability (Vaidya et al., 2024, 2016; Santos et al., 2024).

The main opportunities, limitations, and factors influencing the successful implementation of nano-enabled microbial bioinputs in agricultural systems are summarized in **Figure 1**. Ultimately, nano-enabled microbial bioinputs should not be viewed as a universal replacement for conventional microbial products but rather as a next-generation technological platform whose successful implementation will depend on integrating advances in microbiology, nanotechnology, formulation engineering, biosafety assessment, long-term agronomic validation, and regulatory harmonization. Their long-term contribution to sustainable agriculture will depend less on the development of increasingly complex formulations than on their ability to translate improvements observed under controlled conditions into consistent agronomic, economic, and environmental benefits across diverse agricultural systems.

**Figure 1:**
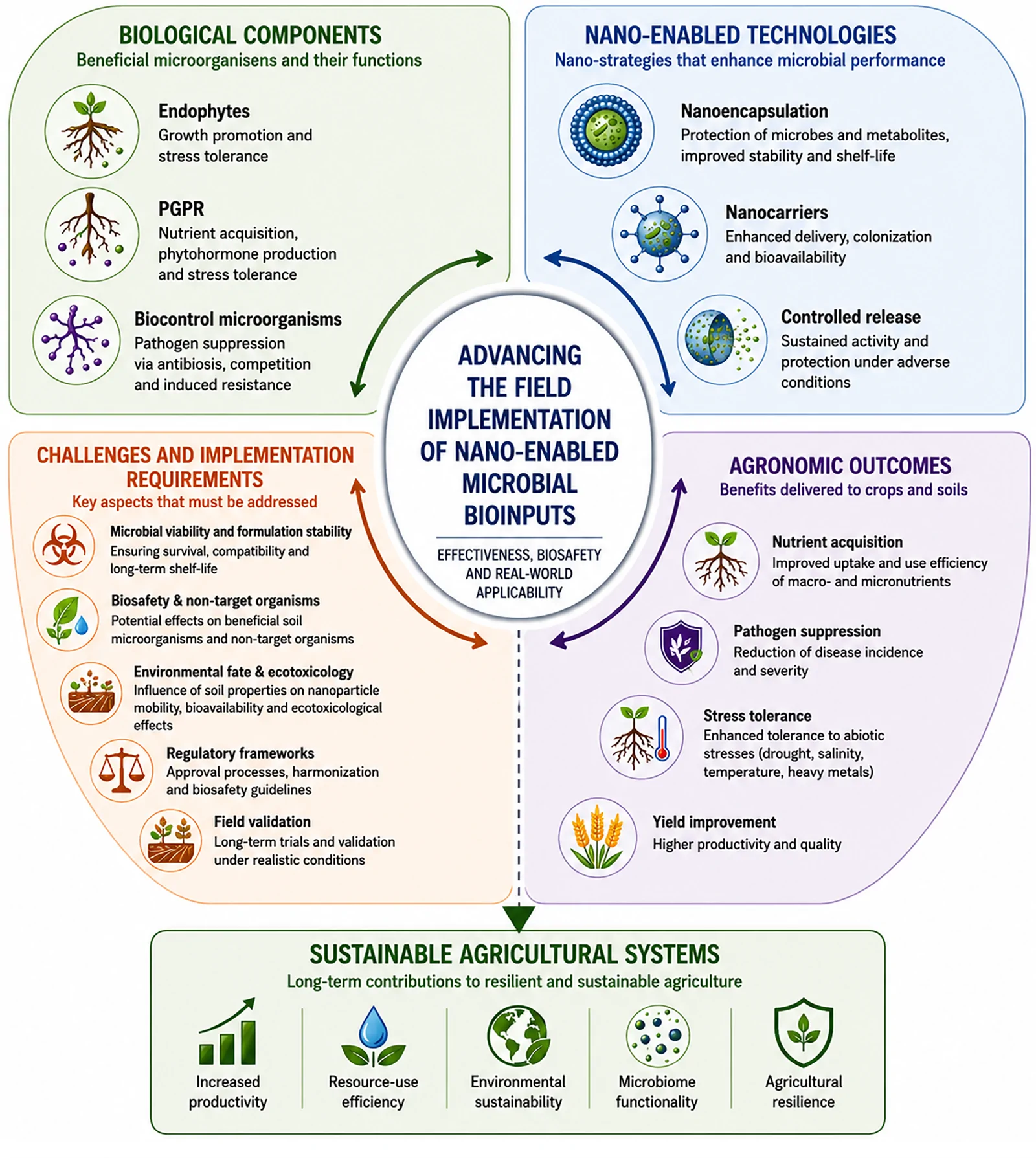
Conceptual framework illustrating how nano-enabled microbial bioinputs improve the field performance of microbial technologies and contribute to sustainable agricultural systems. Beneficial microorganisms and nano-enabled technologies interact to enhance nutrient acquisition, pathogen suppression, stress tolerance, and crop productivity. However, successful implementation depends on addressing challenges related to microbial viability, biosafety, environmental fate, ecotoxicological effects on beneficial and non-target organisms, regulatory approval, and long-term field validation under realistic agricultural conditions.

## 8 Conclusions and future perspectives

The body of evidence presented in this review demonstrates that nano-enabled microbial bioinputs represent a promising technological platform for improving the stability, delivery efficiency, and agronomic performance of microbial bioinputs for plant growth promotion and biological disease control. By integrating advances in microbiology, nanotechnology, and formulation engineering, these technologies offer new opportunities to address several long-standing limitations associated with conventional microbial bioinputs, particularly with respect to microbial persistence, protection of biological components, and the consistency of field performance under agricultural conditions.

Despite these advances, the successful translation of nano-enabled microbial bioinputs into commercially viable agricultural solutions will require overcoming important scientific, technological, and regulatory challenges. Future research should prioritize long-term field validation across diverse agricultural systems, standardized protocols for efficacy, biosafety, and agronomic performance assessment, and a deeper understanding of the complex interactions among nanomaterials, microorganisms, plants, soil properties, native microbiomes, and non-target organisms. Continued advances in formulation engineering, scalable manufacturing processes, regulatory harmonization, and integrated risk assessment frameworks will also be essential to accelerate the transition of these technologies from laboratory research to practical agricultural applications.

Ultimately, the success of nano-enabled microbial bioinputs will depend less on the development of increasingly sophisticated nanoformulations than on their ability to consistently deliver agronomic, environmental, and economic benefits under realistic field conditions. Achieving this goal will require closer integration of microbiology, nanotechnology, microbial ecology, materials science, formulation engineering, and precision agriculture to bridge the gap between laboratory innovation and sustainable field implementation. As these challenges are progressively addressed, nano-enabled microbial bioinputs have the potential to become a next-generation technological platform supporting more resilient, productive, and environmentally sustainable agricultural systems.
